# VO_2_(B) nanobelts/reduced graphene oxide composites for high-performance flexible all-solid-state supercapacitors

**DOI:** 10.1038/s41598-019-47266-6

**Published:** 2019-07-25

**Authors:** Weifeng Lv, Can Yang, Ge Meng, Ruifeng Zhao, Aijuan Han, Rong Wang, Junfeng Liu

**Affiliations:** 1 Department of Polymer Science and Engineering, School of Chemistry and Chemical Engineering, Nanjing University, Nanjing, 210023 China; 2 State Key Laboratory of Enhanced Oil Recovery, Research Institute of Petroleum Exploration & Development, CNPC, Beijing, 100083 China; 3 State Key Laboratory of Chemical Resource Engineering, Beijing University of Chemical Technology, Beijing, 100029 China

**Keywords:** Electrochemistry, Materials for energy and catalysis, Supercapacitors

## Abstract

Vanadium oxide has attracted extensive attention for electrochemical capacitors due to its wide range of versatility. However, due to the relative poor conductivity and chemical stability of vanadium oxide, severe losses of capacitance often occur during charge and discharge processes. Herein, a free-standing vanadium dioxide (VO_2_(B)) nanobelts/reduced graphene oxide (VO_2_/rGO) composite film was fabricated by assembly of VO_2_(B) nanobelts and rGO for supercapacitors. The flexible rGO sheets and VO_2_(B) nanobelts intertwined together to form a porous framework, which delivered a 353 F g^−1^ specific capacitance at 1 A g^−1^, and after 500 cycles, the specific capacitance retention rate was 80% due to the enhanced conductivity of the VO_2_(B) nanobelts by rGO and increased transport of ions and electrons by the porous structures. An all-solid-state symmetrical supercapacitor was assembled from the VO_2_/rGO composites, which exhibited good energy storage performance with a maximum voltage of 1.6 V. The maximum power density is 7152 W kg^−1^ at the energy density of 3.13 W h kg^−1^, ranking as one of the highest power densities for reported materials. In addition, after 10000 cycles, it still has a specific capacitance retention rate of 78% at 10 A g^−1^.

## Introduction

In recent years, the energy crisis caused by the rapid consumption of fossil energy has stimulated people to continuously explore renewable energy and new types of energy storage devices^1^. Among them, electrochemical capacitors (ECs), also known as supercapacitors (SCs), can guarantee operational safety, and have received widespread attention compared with traditional tablet capacitors, secondary batteries, fuel cells, and lithium-ion batteries^2–5^. SCs can provide high power density and good cyclic stability, which can be used in electric vehicles and portable electronic devices. Following the trend of portable and wearable electronics becoming small, lightweight and flexible, many researches on supercapacitors have focused on the study of flexible all-solid-state devices recently^6^.

VO_2_(B), as one of the metastable phases of vanadium dioxide, has attracted increasing attention in energy storage devices. It has layered structure and multi-oxidation states, which facilitates the storage of charge through insertion as well as fast Faradaic reaction on the surface^7–9^. However, the structural instability of VO_2_(B) results in bad cyclic stability^10,11^. Carbon-based materials, like graphene, carbon black and carbon nanotubes, are widely used to improve the electrodes’ conductivity and stability in supercapacitors^12^. Graphene has become one of the most popular materials for supercapacitors compared with other carbon-based materials since its mechanical flexibility, excellent electrical conductivity, thermal stability and chemical stability^13–15^. Nevertheless, because of the agglomeration of graphene, its specific capacitance is much lower than the theoretical capacity, which limits the application of graphene in practice^16,17^. Therefore, heterogeneous nanomaterials composed of multicomponent nanomaterials attract people’s attention, because they can make full use of the advantages of each component to greatly enhance the performance of supercapacitors.

Combining the merits of graphene and VO_2_(B), various VO_2_(B)/carbon composites could be obtained. For example, VO_2_(B)/graphene composites were reported to achieve the specific capacitance of 245 F g^−1^ at 1 A g^−1 ^^18^. A starfruit-like VO_2_(B)/graphene hybrid electrode showed 225 F g^−1^ at 0.25 A g^−1^ in 0.5 M K_2_SO_4_ solution^15^. Core–shell VO_2_(B)/C composites delivered a specific capacitance of 182 F g^−1^ at 1 A g^−1 ^^19^. Nevertheless, as the supercapacitors, the electrochemical property of VO_2_(B) is not satisfactory. Hong *et al*. examined the effect on electrochemical performance of supercapacitors composed of VO_2_(B)/graphene oxide (GO) electrodes due to the transverse dimension of graphene. The hybrid electrode with ultra-large graphene oxide (UGO) performed excellent capacitive performance (769 F g^−1^), however the very low yield of the preparation of UGO limit its practical application^20^. 1D VO_2_(B) nanostructures are semimetal material with great flexibility and large surface area, which make them easily intertwine to paper-like film with little amount of binders in the fabrication of flexible electrodes and promising in ultra-thin flexible devices^21–23^. It adds an additional advantage in preventing breakage of the electrode frame due to mechanical bending/twisting of the flexible device. However, their role as the flexible supercapacitors electrode material in smart portable and wearable electronics is rarely reported so far.

Herein, we fabricated a free-standing VO_2_(B) nanobelts/reduced graphene oxide (VO_2_/rGO) composite film by assembly of rGO and thin vanadium dioxide (VO_2_(B)) nanobelts. As expected, it exhibited superior capacitance of 353 F g^−1^ at 1 A g^−1^. Using VO_2_/rGO composite film electrodes and gel organic electrolyte, the flexible all-solid-state supercapacitor was fabricated. The maximum power density was 7152 W kg^−1^ at the energy density of 3.13 W h kg^−1^. The device has the advantages of large energy density and high power density, and has broad application prospects in flexible energy storage devices.

## Results and Discussion

**Figure 1:**
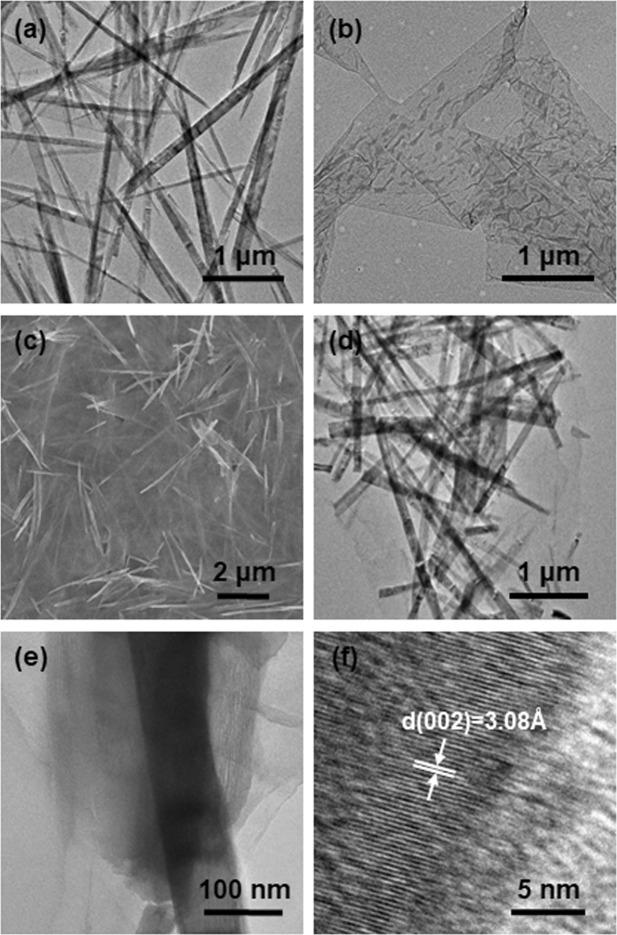
Typical TEM images of (**a**) VO_2_(B) and (**b**) rGO. Typical (**c**) SEM image, (**d**,**e**) TEM images and (**f**)HRTEM image of VO_2_/rGO-2.

The morphologies of VO_2_(B) nanobelts, GO, and VO_2_/rGO composites were characterized by SEM and TEM and shown in Fig. 1 and S1. As can be seen from Fig. 1a, the synthesized VO_2_(B) were composed of uniform nanobelts, with length of about 3–5 μm and width of about 40–150 nm. It indicated the successfully preparation of vanadium dioxide with a long aspect ratio, which is conducive to the rapid electron transfer in the charge/discharge process. The synthesized GO were ultrathin and nearly transparent, with some wrinkles seen from its TEM image (Fig. 1b). In the composites, the VO_2_(B) nanobelts and GO were well mixed and the more GOs there were, the more VO_2_(B) nanobelts were covered (Fig. 1c,d and S1). To further characterize the structure of the VO_2_/rGO composite, high resolution TEM images were taken. The VO_2_(B) nanobelts were covered by the GO (Fig. 1e). A clear lattice fringe with a spacing of 0.308 nm were observed in a typical HRTEM image, which agreed well with the (002) plane spacing of VO_2_(B). The nearby amorphous structure was generated from rGO, and this nanosized mixing indicated that a tight specific binding between VO_2_(B) nanobelts and rGO was achieved. The closely attached composite structure was beneficial to ease the stress due to the volume expansion and contraction of the material in charge-discharge test, which would be favorable to improve the stability of vanadium dioxide in electrochemical measurements.

**Figure 2:**
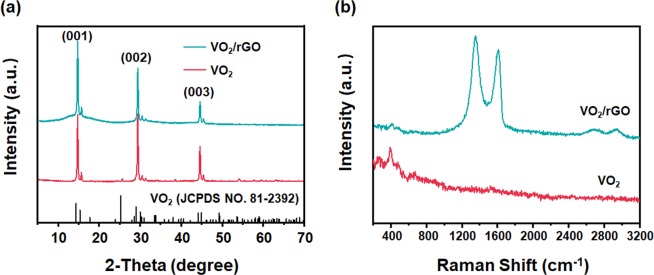
(**a**) XRD patterns and (**b**) Raman spectra of VO_2_(B) nanobelts and VO_2_/rGO-2.

XRD measurement was used to obtain information about the phase composition of the products (Fig. 2a). The pattern of VO_2_(B) nanobelts disclosed three sharp and strong peaks at 2θ of 14.8°, 29.7° and 44.6°, corresponding to the (001), (002) and (003) crystal planes of VO_2_(B) (JCPDS NO. 81–2392). In the XRD pattern of VO_2_/rGO-2 (which means 2 mL GO aqueous dispersion was used to assemble the VO_2_/rGO film) composite, the three main peaks of VO_2_(B) were presented, while a broad peak at 10°–20° from the rGO was also shown. This broad peak became higher with the increasing rGO amount (Fig. S2). It indicated that the two components maintained their phase structure in the mechanical composite film and the composition can be well controlled by tuning the ratio of them in the preparation process. The structure information of the sample was further characterized by Raman spectroscopy (Fig. 2b). In the Raman spectrum of VO_2_(B) nanobelts, three peaks at 278 cm^−1^, 399 cm^−1^, 684 cm^−1^ appeared, which could be assigned to the bending of V-O-V bond, the V-O-O bridge bond, and the three coordination bonds between vanadium atoms and oxygen atoms, respectively^24^. In the Raman spectrum of the VO_2_/rGO composite, the feature peak of VO_2_(B) could be observed at 399 cm^−1^, further testifying the existence of VO_2_(B) nanobelts in the composite. Besides, strong D band (K-point photons of A_1g_ symmetry) peak at 1350 cm^−1^ and G band (the E_2g_ phonon pattern of the sp^2^ carbon) peak at 1590 cm^−1^ arise, indicating the presence of rGO in the composite material. The relative intensity of D and G peaks (I_D_/I_G_) was calculated to be 1.133, indicating that hydrazine hydrate successfully reduced the GO and a high degree of ordered rGO was achieved in the composite.

**Figure 3:**
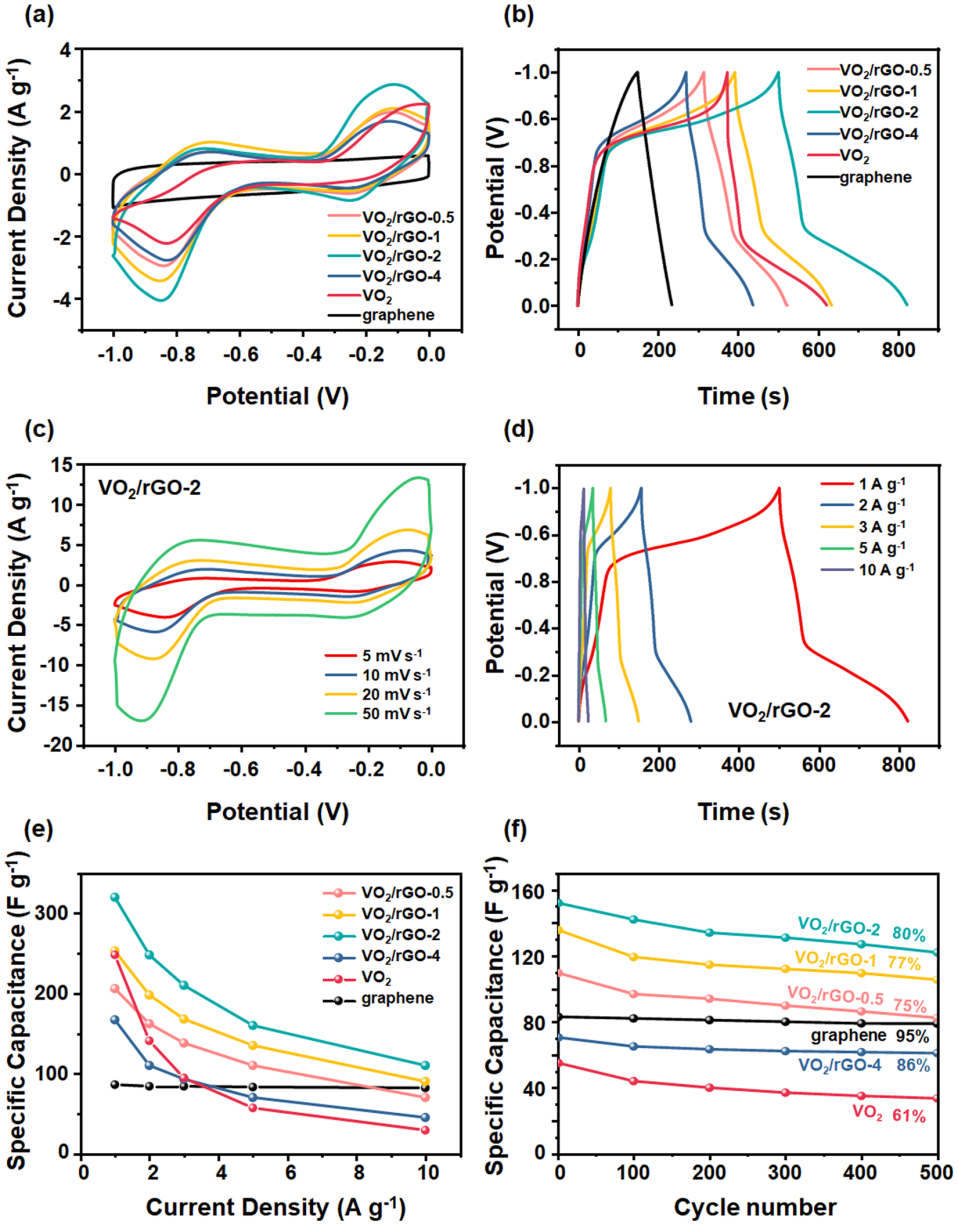
Electrochemical properties of VO_2_/rGO composite**s**, VO_2_(B) nanobelts and rGO. (**a**) CV curves (5 mV s^−1^), (**b**) galvanic charge/discharge time curves (1 A g^−1^), (**c**) CV curves at different scan rates and (**d**) galvanic charge/discharge time curves at different current density of VO_2_/rGO-2. (**e**) Rate capabilities and (**f**) cyclic stability test of VO_2_/rGO-2, VO_2_(B) nanobelts and rGO.

To test the potential of VO_2_/rGO composites as electrode materials for the supercapacitor, the electrochemical measurements were conducted in a three-electrode system with 0.5 M K_2_SO_4_ aqueous solution as the electrolyte. CV measurements were carried out within the potential range from −1.0 to 0 V at 5 mV s^−1^ scan rate, and the results were shown in Fig. 3a. The rGO exhibited a typical rectangular shape CV curve, manifesting that the rGO possesses excellent electrical double-layer capacitance. The VO_2_(B) nanobelts demonstrated two pair of well-defined redox peaks near −0.75 V, −0.15 V and −0.85 V, −0.25 V, which represents the faradaic redox reactions associated to VO_2_/K_x_VO_2_. The process was shown as follows

1 $${{\rm{V}}{\rm{O}}}_{2}+{{\rm{x}}{\rm{K}}}^{+}+{{\rm{x}}{\rm{e}}}^{-}\leftrightarrow {{\rm{K}}}_{{\rm{x}}}{{\rm{V}}{\rm{O}}}_{2}$$

where x is the mole fraction of inserted K^+^ ions. The VO_2_/rGO composites also gained two pair of faradaic redox peaks, but their integrated areas were significantly larger than that of pure rGO and VO_2_(B) nanobelts. Among these composites, VO_2_/rGO-2 composites gave the highest CV integrated area. These results suggested that the VO_2_/rGO composites had higher capacitance than that of the pure compounds, while VO_2_/rGO-2 obtained the highest capacitance. The CV curves at different scan rate of VO_2_/rGO-2 were shown in Fig. 3c, and the current density increased with increasing scan rate from 5 mV s^−1^ to 50 mV s^−1^.

Galvanostatic charge/discharge were also used to study the electrochemical performance of the materials at 1 A g^−1^ between 0 and −1.0 V (Fig. 3b). VO_2_/rGO-2 had the longest discharge time, which means the highest capacitance, in accordance with the above CV curve results. The specific capacitance was calculated by C=I∆t/m∆V, where I refers the discharge current, ∆t is the discharge time from 0 to −1.0 V, m is the mass of the electrode and ∆V is the voltage difference during the discharge time. The maximum specific capacitance of 353 F g^−1^ was obtained at 1 A g^−1^ for VO_2_/rGO-2 composite compared to 80 and 248 F g^−1^ for rGO and VO_2_(B) nanobelts, respectively. This indicated that VO_2_/rGO composites had better electrochemical activity, in consistent with CV results. The superior specific capacitance of the composite over pure components can be attributed to the synergistic effect of enhanced electron transfer originated from graphene, and enhanced charge storage due to the VO_2_(B) nanobelts which was enhanced on the surface of 1D nanobelts than bulk structures^25–29^. Moreover, the galvanic charge/discharge time curves at different current density of VO_2_/rGO-2 are shown in Fig. 3d.

The capacitance retention ratio in the wide current density range is another basic characteristic required for practical applications that meet the high power requirements of supercapacitors. The relationship between current density and specific capacitance for different content of rGO composites was illustrated in Fig. 3e. The specific capacitance of the VO_2_/rGO-2 electrode was higher than the other contrast samples over the entire current density range. The capacitance retention for VO_2_/rGO-2 composite was 33.1% from 1 A g^−1^ to 10 A g^−1^. In contrast, the pure VO_2_(B) nanobelts showed much poor rate capability (only 10.9% retained from 1 A g^−1^ to 10 A g^−1^). It indicated that the rational combination of rGO and VO_2_(B) could effectively improve the capacitances.

The cycling stability of electrodes was evaluated by charge/discharge process at 5 A g^−1^. The specific capacitance of rGO, VO_2_(B) nanobelts and VO_2_/rGO composites in 500 cycles were shown in Fig. 3f. After 500 cycles, the specific capacitance of VO_2_(B) nanobelts dropped to 61% while the rGO still maintained 95%. The VO_2_/rGO composites electrode demonstrated remarkable cycling stability than the pure components, and the higher the graphene in the composites, the more stable the electrode was. The VO_2_/rGO-2 gained a capacitance retention rate of approximately 80% after 500 cycles, which was superior to the performance of VO_2_(B) nanobelts. The significant attenuation of the specific capacitance may be due to ion insertion/deintercalation induced material pulverization and chemical dissolution^29^. The results showed that the combination of vanadium oxide and rGO can increase the electrochemical stability of the VO_2_(B) nanobelts electrode.

**Figure 4:**
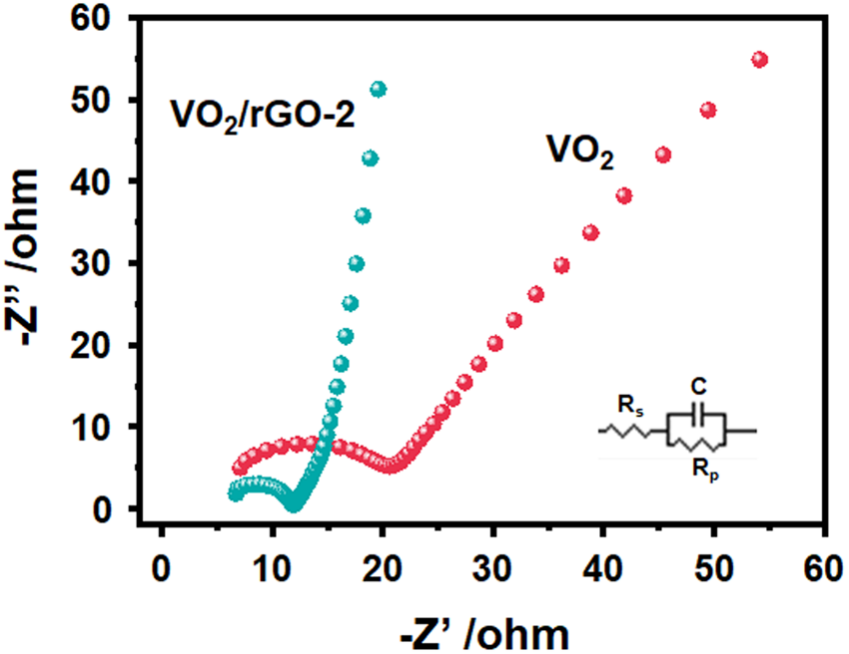
EIS spectra of VO_2_(B) nanobelts and VO_2_/rGO-2.

Electronic impedance spectra (EIS) were also used to study the electrical properties of the electrode materials. Figure 4 showed the complex-plane impedance plots for VO_2_(B) nanobelts and VO_2_/rGO-2 at 0 V, where in the Nyquist plot, the real (Z′) and imaginary (Z″) components gave the frequency dependent impedance. Both graphs were of semicircular shapes in high frequency regions caused by the charge transfer process of the Faradaic reaction, and followed by a relatively linear response in the low frequency region. It was found that the VO_2_/rGO-2 had a similar series resistance to VO_2_(B) nanobelts. From Fig. 4, the charge transfer resistance increased from VO_2_(B) nanobelts to VO_2_/rGO-2. Meanwihle, through circuit simulation, the obtained R_ct_ values were 16.1 and 5.9, corresponding to VO_2_(B) nanobelts and VO_2_/rGO-2, respectively. VO_2_/rGO-2 has interconnected porous networks compared with VO_2_(B) nanobelts, which means electrolyte ions and electrons can easily diffuse through the interface between VO_2_ and rGO framework, resulting superior electrochemical activity and stability.

**Figure 5:**
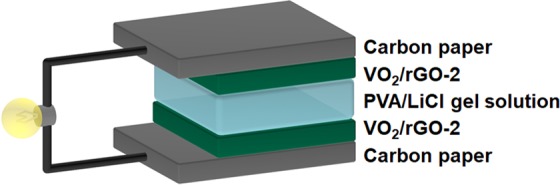
Schematic illustration of the all-solid-state supercapacitor.

**Figure 6:**
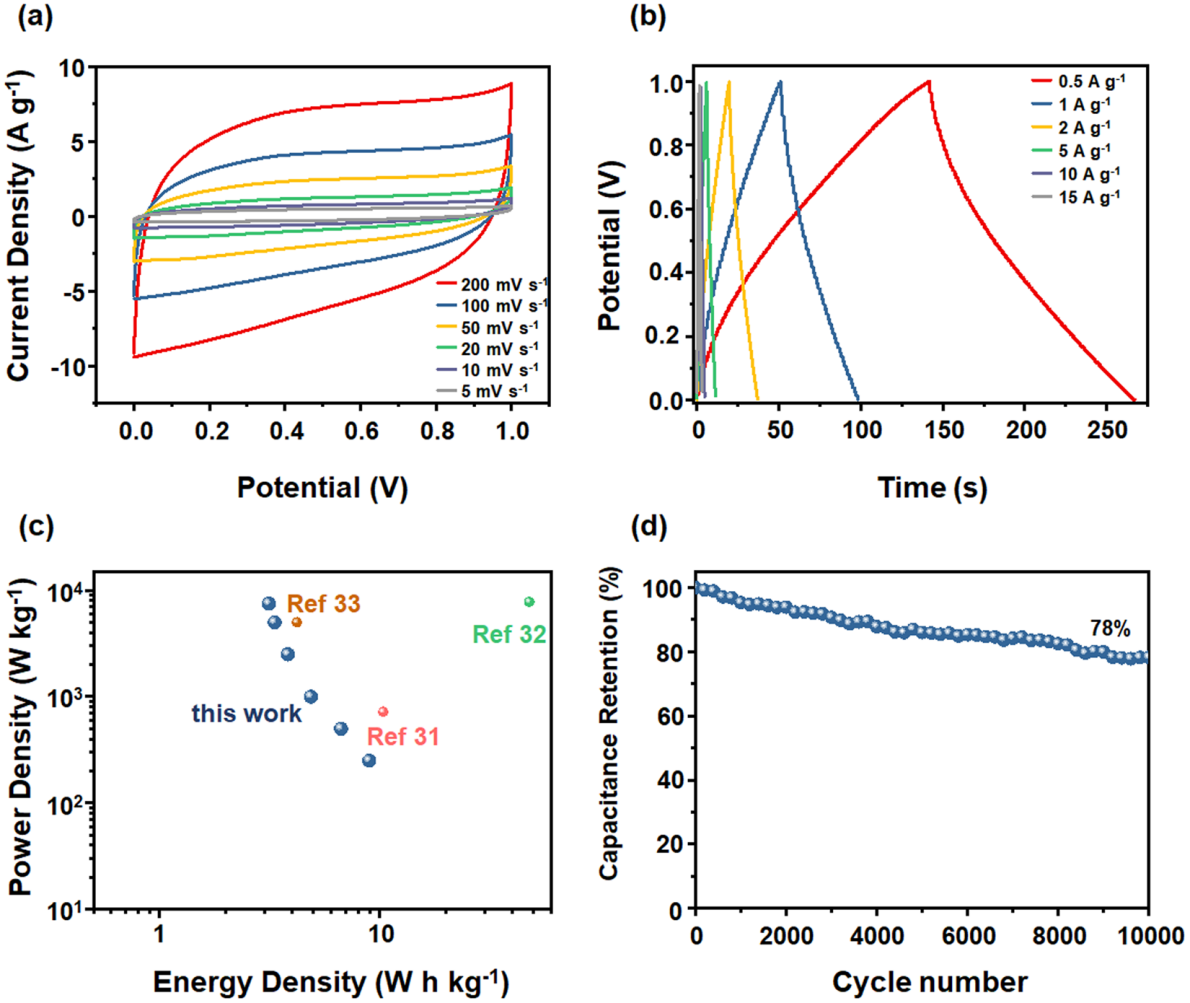
VO_2_(B)/rGO-2 based all-solid-state supercapacitors. (**a)** Cyclic voltammetry curves at different scan rates, (**b**) galvanostatic charge/discharge curves at different current densities, (**c**) Ragone plot and (**d**) cyclic stability test.

All-solid-state supercapacitors have engaged widespread attention in recent years due to their lightweight, small size, environmental protection, and portability. Figure 5 gave the assembly schematic diagram of a VO_2_/rGO-2 composite all-solid-state supercapacitor. We cut the VO_2_/rGO-2 composite into two pieces with the same area, which were used as the cathode and anode respectively. Carbon paper was used as current collectors and PVA/LiCl as gelled electrolyte to assemble an all-solid-state supercapacitor. The electrochemical measurements were proceeded, and the results were demonstrated in Fig. 6. Figure 6a showed the cyclic voltammogram curves of the device at different scan rates. When the scan rate changed from 5 to 200 mV s^−1^, the area of the cyclic voltammogram curves gradually increased, and the shape almost didn’t not change, demonstrating the excellent fast charge and discharge characteristics of the device. In order to further measure the electrochemical performance of the device, a galvanostatic charge/discharge test was proceeded (Fig. 6b), and the active substance loading was 0.5 mg cm^−2^. At 1 A g^−1^, the specific capacitance of the all-solid-state supercapacitor was calculated to be 29.6 F g^−1^. Meanwhile, as the current density increased, the capacity of the all-solid-state supercapacitor didn’t attenuate much, exhibiting good rate capability. The relationship between specific power and specific energy was illustrated in Fig. 6c. The power density and energy density were calculated grounded on the total mass of the cathode and anode electrodes of the device. The maximum energy density was 8.96 W h kg^−1^ at the power density of 250 W kg^−1^, while the maximum power density of 7512 W kg^−1^ was obtained at the energy density of 3.13 W h kg^−1^. The performance of our all-solid-state supercapacitors were compared with reported materials (Fig. 6c and Table S1), and its power density was among the highest values^30–33^. In order to measure the cycling stability of the device, the all-solid-state supercapacitor was conducted to a continuous charge/discharge test at 10 A g^−1^ (Fig. 6d). After 10000 cycles, the capacity remained 78% compared with that before the cycling, indicating that compared to the aqueous electrolyte, the gel electrolyte can improve the stability of the device.

## Conclusions

In summary, the combination of vanadium dioxide and rGO can improve the conductivity and stability of the VO_2_(B) nanobelts. The flexible rGO sheets and the vanadium dioxide nanobelts were interconnected to form a porous network structure, which facilitated the transport of ions and electrons. Besides, it can improve the conductivity of the VO_2_(B) nanobelts, resulting in the improved electrochemical performance of the material. When used as supercapacitor electrodes, the VO_2_/rGO composite exhibited remarkable rate capability, high specific capacitance and excellent cycling stability. What’s more, when the composite material was assembled into an all-solid-state supercapacitor, excellent electrochemical performance was exhibited.

## Materials and Methods

### Chemicals

Graphite powder (analytical grade) were purchased from Aladdin. Sodium nitrate (NaNO_3_), potassium permanganate (KMnO_4_) and ammonium metavanadate (NH_4_VO_3_) were of analytical grade and purchased from Sinopharm. Formic acid (HCOOH, 99%) was purchased from Guangfu Fine Chemicals. Phosphoric acid (H_3_PO_4_, 99%), sulfuric acid (H_2_SO_4_, 95~98%), hydrogen peroxide (H_2_O_2_, 30%), hydrochloric acid (HCl, 36~38%), potassium sulfate (K_2_SO_4_) and hydrazine hydrate (N_2_H_4_·H_2_O, 80%) were purchased from Beijing Chemical Works. All the chemicals were used as received without further purification.

### The preparation of VO_2_/rGO composites

Graphene oxide (GO) and VO_2_(B) nanobelts were firstly prepared by modified Hummers method and hydrothermal method respectively (the details were presented in the Supplementary Information)^6^. In a typical procedure for three-dimensional (3D) VO_2_/rGO composites, a certain volume of GO aqueous dispersion (5 mg mL^−1^) was added into 30 mL deionized water and sonicated for 30 min, and then mixed with 3 mL VO_2_(B) ethanol dispersion (5 mg mL^−1^) under magnetic stirring for 30 min. Subsequently, 0.2 mL hydrazine hydrate (80 wt%) was added into the above suspension under magnetic stirring for 30 min. The precipitate was then washed several times with deionized water to remove the residue. The mixture was filtered by using facile vacuum filtration and dried in air. Finally, the product was carefully peeled off from the filter paper. Samples with different contents of graphene were prepared by changing the amount of GO and denoted as VO_2_/rGO-x (x means the volume of GO aqueous dispersion of 4 mL, 2 mL, 1 mL and 0.5 mL used to assemble the VO_2_/rGO film).

### Material characterization

The composition and morphology of the products were carried out by a field-emission scanning electron microscope (SEM) (Zeiss SUPRA 55) operating at 20 kV. The structure and size of the products were characterized using a transmission electron microscopy (TEM) system (FEI Tecnai G^2^ 20 S Twin). Raman spectroscopy using a wavelength of 514 nm was collected with RM100 under ambient conditions, and the laser spot size was about 1 μm. Powder X-ray diffraction (XRD) patterns were recorded on a Shimadzu XRD-6000 diffractometer with Cu Kα radiation (λ = 1.54056 Å) in the 2θ range from 5° to 70° at a scan rate of 5°/min.

### Electrode material performance measurements

Electrodes were prepared by mixing the VO_2_/rGO composite, acetylene black, perfluorosulfonic acid-polytetrafluoroethylene copolymer (Nafion, 5 wt%) in a mass of 5 mg, 0.625 mg and 0.625 mg, respectively, and dissolved in absolute ethanol to obtain 1 mL slurry. Then 5 μL of the slurry was coated onto the surface of the glassy carbon electrode and dried with an infrared lamp. The loading mass of the active substance was approximately 0.025 mg. Electrochemical testing of individual electrodes was performed in a three-electrode system with 0.5 M K_2_SO_4_ aqueous solution as electrolyte. Saturated calomel electrode (SCE) and Pt foil were used as reference and counter electrode, respectively.

### All-solid-state supercapacitor assembly and electrochemical measurements

The method of preparing the LiCl/PVA gel electrolyte was as follows. 3.0 g of polyvinyl alcohol (PVA) powder was added into 30 mL of deionized water under magnetic stirring at 95 °C in a water-bath until the PVA powder completely dissolved. Then 1.25 g of LiCl was added to the PVA solution and stirred vigorously until a homogeneous viscous solution was formed. The solution cooled naturally to room temperature to get a clear transparent gel electrolyte. The all-solid-state supercapacitor was prepared by tiling two pieces of the same sized VO_2_/rGO electrodes (1.0 cm × 1.0 cm) on carbon paper collectors (1.0 cm × 2.0 cm), and covering one of them with a LiCl/PVA gel electrolyte layer. The paper collector was dried at room temperature for 30 min. The semi-dried electrodes were then put face-to-face and finally the edge of the supercapacitor was sealed with polyethylene terephthalate (PET).

## Supplementary information

Supplementary Information with changed mark

Supplementary Information
