# Supplementary material for: VO2(B) nanobelts/reduced graphene oxide composites for high-performance flexible all-solid-state supercapacitors
Source: Sci Rep. 2019 Jul 25;9:10831. doi: 10.1038/s41598-019-47266-6 (PMC6658522; doi:10.1038/s41598-019-47266-6)
Supplement: Supplementary file 1 — Supplementary Information with changed mark [file 41598_2019_47266_MOESM1_ESM.docx]

**SUPPLEMENTARY INFORMATION**

**VO_2_(B) nanobelts/reduced graphene oxide composites for high-performance flexible all-solid-state supercapacitors**

Weifeng Lv^1,2§^, Can Yang^3§^, Ge Meng^3^, Ruifeng Zhao^3^, Aijuan Han^3*^, Rong Wang^1*^, Junfeng Liu^3^

^1^Department of Polymer Science and Engineering, School of Chemistry and Chemical Engineering, Nanjing University, Nanjing 210023, China. ^2^State Key Laboratory of Enhanced Oil Recovery, Research Institute of Petroleum Exploration & Development, CNPC, Beijing 100083, China. ^3^State Key Laboratory of Chemical Resource Engineering, Beijing University of Chemical Technology, Beijing 100029, China. ^§^These authors contributed equally to this work. Correspondence and requests for materials should be addressed to R.W. (email: rong_wang2001@163.com) or A.H. (email: hanaijuan@mail.buct.edu.cn).

**Synthesis of graphene oxide**

A typical synthesis procedure is as follows: Graphite powder (1 g) was placed in a mixture of concentrated sulfuric acid (108 mL) and concentrated phosphoric acid (12 mL). Potassium permanganate (3 g) was gradually added while stirring, while keeping the liquid mixture below 5° C by a cold water bath. The mixture was then stirred for three days and then added with 30% hydrogen peroxide (10 mL). After one hour’s standing, 100 mL of deionized water was added to the mixture, and the graphene was gradually oxidized to obtain graphene oxide. After the temperature of the mixture was lowered to room temperature, the mixture was centrifuged and the precipitate was washed with a 10% HCl solution to remove residual metal ions. The precipitate was then washed with deionized water until it became neutral. Finally, the cleaned precipitate was dispersed in 200 mL of deionized water. The concentration of the graphene oxide dispersion was 5 mg/mL.

**Synthesis of vanadium oxides nanobelts**

0.234 g NH_4_VO_3_ was dispersed in 20mL deionized water, and 0.4mL HCOOH was added to the above suspension. Then, the suspended orange red solution was transferred into a 40mL Teflon-lined autoclave and was hydrothermally treated at 180℃ for 48 h. The precipitate was washed with distilled water and absolute ethanol three times, respectively. The obtained VO_2_(B) nanobelts were dispersed in 20 mL ethanol, and the concentration was 5 mg/mL.


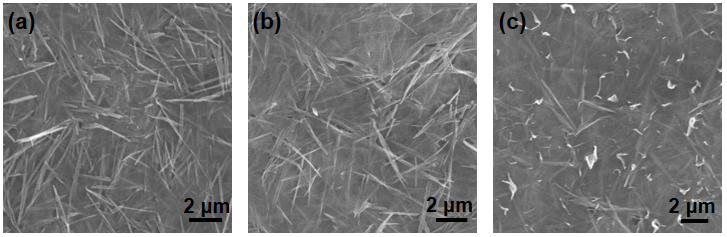


**Figure S1.** Typical SEM images of VO_2_/rGO composites with the different graphene contents: (a) VO_2_/rGO-0.5; (b) VO_2_/rGO-1; (c) VO_2_/rGO-4.


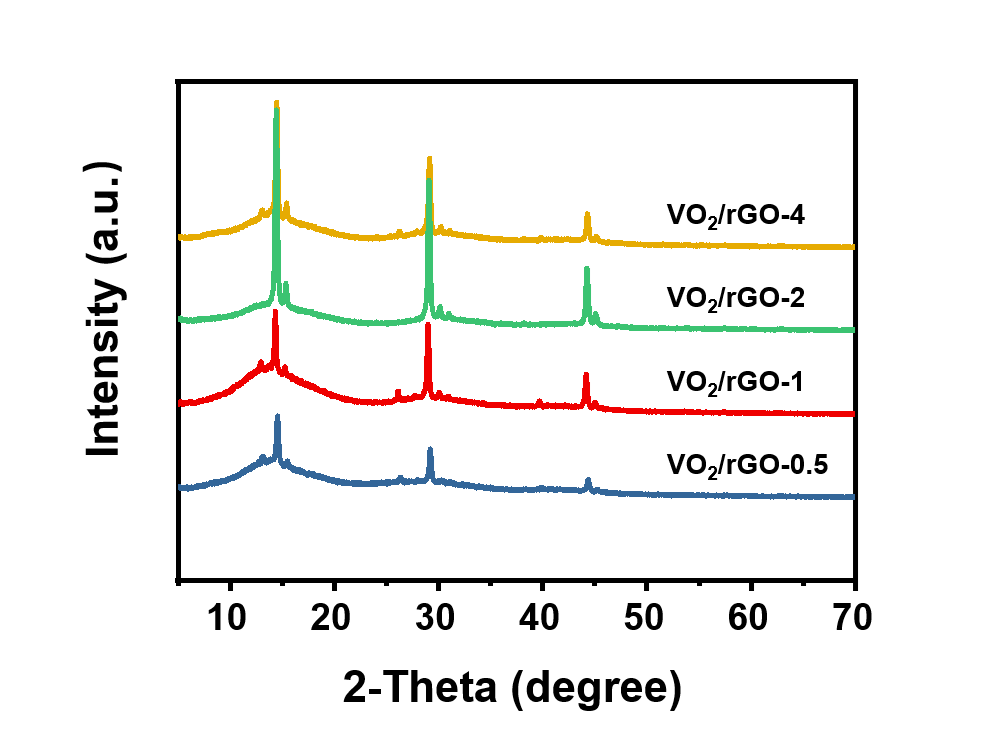


**Figure S2.** XRD patterns of VO_2_/rGO composites with the different graphene contents.

| Supercapacitors | Energy Density(Wh kg^-1^) | Power Density (kW kg^-1^) | Electrolyte | **Ref.** |  |
| --- | --- | --- | --- | --- | --- |
| 86.2 % V_6_O_13_ 13.8 % VO_2_//86.2 % V_6_O_13_ 13.8 % VO_2_ | 22.8  (at 0.18 kW kg^-1^) | 1.2  (not mentioned) | 1 M LiNO_3_ | J. Mater. Chem. A 2015, 3, 22892. |  |
| NiCo_2_S_4_@NiO//AC | 30.385  (at 0.288 kW kg^-1^) | 0.72  (at 10.36 Wh kg^-1^) | PVA-KOH | Sci. Rep. 2016, 6, 38620. |  |
| Zn-Co-S NWs//Fe_2_O_3_@rGO | 81.6  (at 0.559 kW kg^-1^) | 7.8  (at 47.9 Wh kg^-1^) | PVA-KOH | Adv. Energy Mater. 2018, 15, 1702014. |  |
| pErGO@Cuf/Cu wire | 11.25  (at 0.25 kW kg^−1^) | 5  (at 4.2 Wh kg^−1^) | PVA/H_3_PO_4_ | Sci. Rep. 2018, 8, 640. |  |
| VO_2_/rGO// VO_2_/rGO | 8.96  (at 0.25 kW kg^-1^) | 7.51  (at 3.13 W h kg^-1^) | PVA/LiCl | This work |  |

**Table S1.** Summary of performance of all-solid-state supercapacitors.
